# Need for Engagement in Stroke Prevention Shared Decision-Making in Older Adults with Atrial Fibrillation and Multimorbidity

**DOI:** 10.20900/agmr20250016

**Published:** 2025-07-24

**Authors:** Hawa O. Abu, Jane S. Saczynski, Michelle Nabi, Annie Ferris, Mayra Tisminetzky, Jerry H. Gurwitz, Kathleen Mazor, Robert J. Goldberg, David Dosa, Alok Kapoor, Daniel Matlock, David D. McManus

**Affiliations:** 1 Division of Geriatric Medicine, Department of Medicine, University of Massachusetts Chan Medical School, Worcester, MA 01605, USA; 2 Department of Pharmacy and Health Systems Sciences, School of Pharmacy, Northeastern University, Boston, MA 02115, USA; 3 Department of Medicine, UMass Chan Medical School, Worcester, MA 01655, USA; 4 Department of Neurology, UMass Chan Medical School, Worcester, MA 01655, USA; 5 Division of Health Systems Science, Department of Medicine, University of Massachusetts Chan Medical School, Worcester, MA 01655, USA; 6 Department of Population and Quantitative Health Sciences, University of Massachusetts Chan Medical School, Worcester, MA 01655, USA; 7 Division of Geriatric Medicine, Department of Medicine, University of Colorado School of Medicine, Aurora, CO 80045, USA; 8 VA Eastern Colorado Geriatric Research Education and Clinical Center, Denver, CO 80045, USA

**Keywords:** atrial fibrillation, multimorbidity, stroke prevention, shared decision-making, older adults

## Abstract

**Aim:**

To assess the prevalence of older adults with AF and multimorbidity expressing a need for greater involvement in SDM for stroke prevention and associated patient characteristics.

**Methods:**

A prospective cohort study (2016–2018) enrolled patients aged 65 years and older with AF from clinics in MA and Georgia. Participants with one or more chronic conditions were included in this study. To assess patient preferences for greater engagement in SDM, participants on oral anticoagulants were asked at the one-year follow-up if they would like to be more involved in deciding to take an anticoagulant and which oral anticoagulant to use for stroke prevention. Multivariable logistic regression analysis was used to identify patient characteristics associated with preference for more SDM engagement.

**Results:**

Among participants (*n* = 532; mean age: 75 years; 48% women, 87% White), 41% had 1–4 chronic conditions, 40% had 5–7, and 19% had 8 or more. Approximately one-third expressed a preference for both engaging in SDM on anticoagulation initiation and choosing the type of anticoagulant for stroke prevention. After multivariable adjustment, participants who were younger (aged 65–74 years), women, non-White, had less than high school education, higher perceived burden from anticoagulation use, or had fewer comorbidities, were more likely to report the need for greater SDM engagement for stroke prevention.

**Conclusions:**

Clinicians should recognize the specific needs of older patients with AF and multimorbidity that seek greater involvement in SDM for stroke prevention. Providing tailored interventions can enhance stroke prevention decision-making in this vulnerable population.

## INTRODUCTION

AF is a significant cardiovascular epidemic of the 21st century, affecting approximately 59 million people worldwide with 3–5 times substantial increase in stroke risk [1–3]. In 2019, 10–15% of ischemic strokes were attributed to AF [4]. The incidence of AF rises with increasing age, disproportionately affecting adults aged 65 years and older who experience the highest burden of AF-related morbidity and mortality [3]. Additionally, the prevalence of multiple chronic conditions is high in older adults with AF, affecting 80–90% of this population [6,7]. Multimorbidity, defined as the presence of two or more chronic conditions, complicates AF care with conflicting therapeutic recommendations that may not align with patient goals of care, leading to lower uptake of recommended therapies, increased healthcare costs, and poorer clinical outcomes [8,9].

In managing non-valvular AF, oral anticoagulation, with warfarin or direct acting oral anticoagulants is the primary strategy for stroke prevention (Class I, Level evidence A recommendation) [10,11]. Despite the effectiveness of oral anticoagulation, only 60% of eligible patients receive anticoagulation for stroke prophylaxis [12]. Older adults with AF and multimorbidity face complex decisional challenges regarding initiating anticoagulation, the choice of oral anticoagulant, and continuing anticoagulation, while considering their underlying multimorbidity, frailty burden, fall risk, decreased renal clearance, and risk of major bleeding [13,14]. Furthermore, choosing anticoagulation can be complicated by healthcare access, the need for monitoring therapeutic anticoagulation levels, and dependence on caregivers for support. Additionally, women with AF are typically older and have a greater burden of comorbidities and a higher risk of stroke compared to men. Despite this, women are less likely to be prescribed oral anticoagulation, particularly vitamin K antagonist in comparison to their male counterparts [15]. Older adults with multimorbidity require a coordinated and holistic approach that recognizes patient preferences and goals in stroke prevention decision-making.

SDM is a guideline-recommended approach by the AHA, ESC, and HRS for optimizing stroke prevention decisions [10,11]. While SDM improves patients’ understanding of treatment risks and benefits, and reduces decisional conflict, its application in older adults with multimorbidity is limited. Several SDM tools and decision aids have been developed to guide anticoagulation therapy for stroke prevention in AF [16–20]. However, significant gaps exist in understanding the needs of older adults with AF and multimorbidity in engaging in stroke prevention decision-making. Addressing these gaps may inform the design of more culturally sensitive and user-friendly approaches for improving SDM engagement in this vulnerable population.

Using data from the SAGE-AF study [21,22], we examined patient need for greater engagement in SDM when initiating anticoagulation and selecting an oral anticoagulant. We further aimed to address the inquiry: Among older adults with AF and multimorbidity, what factors are associated with a preference for greater engagement in SDM when initiating anticoagulation therapy and choosing an oral anticoagulant?

## METHODS

### Study Population

We utilized data from SAGE-AF multicenter prospective cohort study, previously described in detail [21,22]. Between 2016 and 2018, participants were recruited from clinics in Central and Eastern MA and Central Georgia, United States. In Central MA, recruitment occurred at four sites: University of Massachusetts Memorial Health Care internal medicine, cardiology, and electrophysiology clinics; and Heart Rhythm Associates of Central MA. In Eastern MA, participants were recruited from Boston University cardiology clinic. Central Georgia recruitment occurred at two sites: Family Health Center and Georgia Arrhythmia Consultants. Eligibility criteria included: (i) age ≥ 65 years; (ii) AF diagnosis confirmed by Holter monitoring, electrocardiography, or EMR documentation of AF; and (iii) a CHA2DS2-VASc risk score ≥ 2. Exclusion criteria: inability to provide informed consent or attend follow-up visits, dementia diagnosis, non-English speaking, incarceration, scheduled invasive procedures with high bleeding risk, other indications for anticoagulation (e.g., mechanical heart valve, pulmonary embolism, deep venous thrombosis), or absolute contraindications to anticoagulation. A total of 1244 patients met the eligibility criteria and were enrolled at baseline [21,22].

Trained research staff obtained data from EMRs at study sites. In-person or telephone interviews were conducted with participants at enrollment and follow-up visits. Ethical approval was granted by the Institutional Review Boards of the University of Massachusetts Medical School, Boston University, and Mercer University. All participants provided written informed consent prior to enrollment.

### Assessment of Multiple Chronic Conditions

Eighteen chronic conditions were identified from participants’ EMRs based on the US-DHHS Strategic Framework on Multiple Chronic Conditions [23]. From the US-DHHS list of 20 conditions, we excluded those with low prevalence in our study or not consistently documented in EMRs, including autism (*n* = 0), human immunodeficiency viral infection (*n* = 0), schizophrenia (*n* = 0), illicit drug use (*n* = 11), and liver disease (*n* = 31). The eighteen conditions were hypertension, dyslipidemia, arthritis, heart failure, anemia, cancer, chronic kidney disease, diabetes mellitus, chronic lung disease, valvular heart disease, depression, anxiety, cardiomyopathy, hypothyroidism, myocardial infarction, angina, peripheral vascular disease, and stroke. These conditions were categorized into three multimorbidity burden subgroups (1–4, 5–7, and ≥8 chronic conditions) based on our sample distribution of chronic conditions and consistent with prior publication describing multimorbidity burden in the SAGE-AF population [7].

### Assessing Patient Preferences for Increased Engagement in SDM for Stroke Prevention

To assess patient’s need for increased engagement in stroke prevention decision-making, participants on oral anticoagulation were asked at one-year of study follow up two questions by telephone interviews: “Would you like to be more involved in deciding to choose to take an anticoagulant?” and “Would you like to be more involved in deciding which anticoagulant to take?”. Affirmative responses to both items were indicative of a need for more SDM involvement in choosing to be on anticoagulation and deciding on the oral anticoagulant for stroke prophylaxis. Both items had a high internal consistency with Cronbach alpha of 0.83 [24].

### Baseline Participant Characteristics

Participant sociodemographic data including age, sex (women vs men), race/ethnicity, and level of education were obtained from EMR, telephone, or face-to-face interviews at enrollment.

Structured interviews at baseline were used to assess social support, self-rated health, frailty, IADLs, visual, hearing, and cognitive impairment. Social support was measured with the 5-items Medical Outcomes Social Support Survey Instrument [25]. Participant’s self-rated health was evaluated with a validated single-item question with responses on a 5-point Likert scale: “In general, would you say your health is excellent, very good, good, fair, or poor?” [26]. The CHS frailty scale assessed frailty with scores 0: not frail, 1–2: pre-frail, and ≥3: frail [27]. The IADLs characterized participants’ abilities with transportation, meal preparation, shopping, housework, managing medications and personal finances [28]. The 30-item MoCA battery evaluated cognitive impairment with scores ranging from 0 to 30 (a cutoff of ≤23 suggestive of cognitive impairment) [29]. Visual and hearing impairment were self-reported by participants.

Clinical characteristics including AF type (paroxysmal, persistent, or permanent), prior ablation therapy, AF treatment approach (rate versus rhythm), and anticoagulation therapy, were obtained from EMRs. Calculated risk scores including CHA2DS2-VASc and HASBLED were derived from medical history in EMRs [30]. PEPPI questionnaire assessed confidence in patient-provider interaction, those with a score ≥45 were classified as being very/extremely confident [31]. ACTS questionnaire was used to assess anticoagulation burden and benefit scores [32].

### Statistical Analysis

The analytic sample included participants during one year of follow-up who were on oral anticoagulation and provided responses to the two items assessing the need for SDM engagement for stroke prevention. Descriptive statistics were used to examine participant’s characteristics according to their preference for SDM engagement initiating and choosing oral anticoagulants. Continuous variables were summarized as means and standard deviations. Categorical variables were reported as proportions. Logistic regression models estimated the unadjusted and multivariable adjusted odds ratios (ORs) with accompanying 95% confidence intervals (CIs). For multivariable adjustment, the choice of confounding variables was based on clinical judgement and statistical significance (*p* < 0.05). Separate logistic regression models were used to analyze factors associated with patient need for more SDM engagement for initiating anticoagulation (Model 1) and choosing the oral anticoagulant type (Model 2) respectively. In Model 1 and Model 2, we created a binary outcome categorized as “Yes-Indicating greater patient need for SDM engagement” and “No-Indicative of no need for more SDM engagement”. In regression Model 1, age, race, education, confidence in patient-provider interaction, perceived anticoagulation benefit and burden were adjusted for in the analysis. In regression Model 2, age, race, education, self-rated health, and perceived anticoagulation benefit and burden were adjusted for in the analysis. To understand if the need for greater engagement in SDM differed between men and women, we performed a sensitivity analysis stratified by sex. In addition, to examine potential differences based on multimorbidity burden, we conducted a sensitivity analysis according to the three multimorbidity groups (1–4, 5–7, and ≥8 chronic conditions). All statistical analyses were done using STATA 18 (StataCorp, College Station, Texas). Model results are shown as ORs and accompanying 95% CI.

## RESULTS

### Baseline Characteristics

Participants in the analytic sample (*n* = 532) were younger (*p* = 0.02), more likely to be men (*p* = 0.03), independent of their IADLs (*p* < 0.001), less likely to be cognitively impaired (*p* < 0.01) or to be frail (*p* = 0.03), compared to those excluded (*n* = 712). Of the included participants (mean age: 75 years, 48% women, 87% White), one-quarter reported low social support, three-quarters had internet access in the preceding four weeks, one-third had visual, hearing, and cognitive impairment. One in ten participants were frail (Table 1). Two-thirds were diagnosed with paroxysmal AF and were on rhythm control. One-half of study participants were on warfarin and 40% were on DOACs. Most participants reported being very/extremely confident in their patient-provider interaction (Table 1).

### Burden of Multiple Chronic Conditions

**Figure 1.**
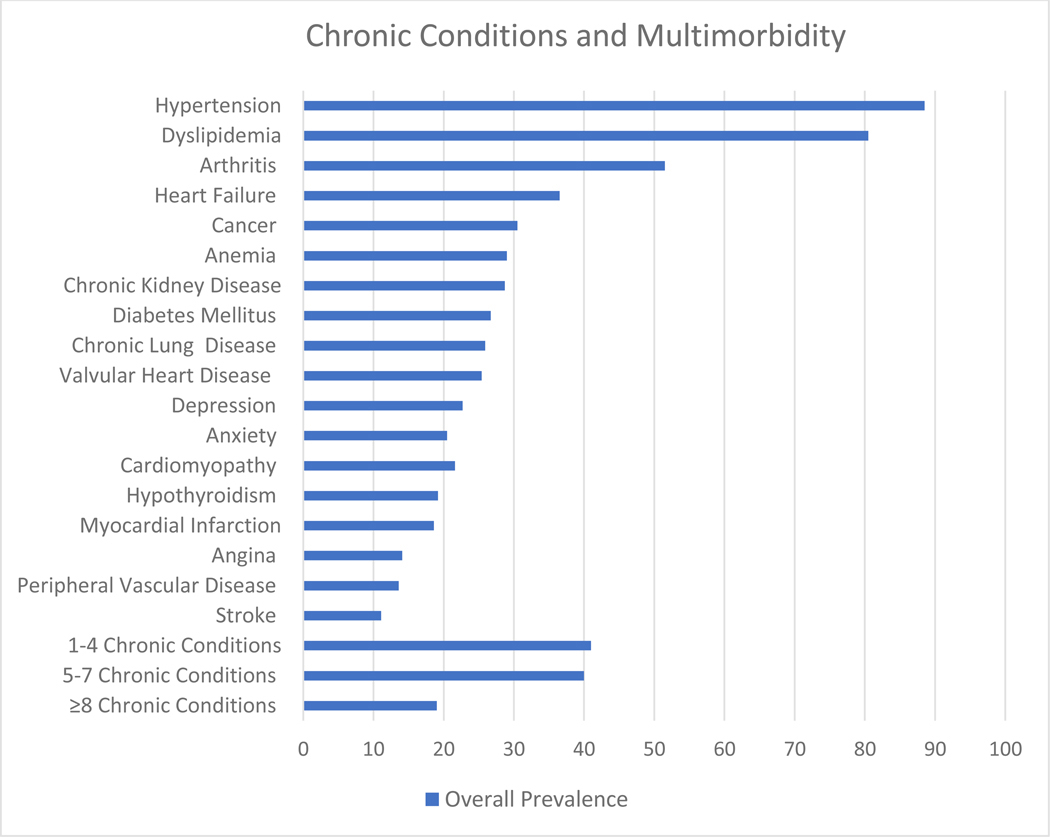
Prevalence of chronic conditions and multimorbidity among older adults with AF in SAGE-AF.

The most prevalent chronic conditions included hypertension (88%), dyslipidemia (80%), and arthritis (51%). Overall, 41%, 40%, and 19% had 1–4, 5–7, and 8 or more chronic conditions respectively (Figure 1).

### Preference for More Engagement in SDM and Associated Patient Characteristics

Approximately one-third of participants expressed a need for greater engagement in SDM on both initiating anticoagulation (*n* = 186; 35%) and selecting the oral anticoagulant (*n* = 175; 33%). Participants who were younger (aged 65–74 years), non-White, and those with a higher perceived burden from anticoagulation use were more likely to have greater need for engaging in SDM on initiating anticoagulation. Conversely, those who were very/extremely confident in their patient-provider interactions and those with higher perceived benefit from anticoagulation use were less likely to report a need for more engagement in SDM for anticoagulation initiation (Table 2).

Regarding engagement in SDM on choosing the oral anticoagulant type, those who were younger (65–74 years), Non-White, had poor self-rated health, and higher perceived anticoagulation burden were more likely to indicate a greater need for more engagement in SDM. Additionally, participants with lower perceived benefit from anticoagulation use were more likely to indicate preference for more engagement in choosing the type of oral anticoagulant for stroke prevention. Overall, no differences in the need for engagement in SDM were observed based on the use of anti-arrhythmic therapy (Table 3).

After adjusting for multiple variables of clinical and statistical significance, participants who were younger (aged 65–74 years) and those with a higher perceived burden from anticoagulation use were more likely to express greater need for more engagement in SDM for initiating anticoagulation (Table 4) and greater engagement in SDM in choosing the anticoagulant (Table 5) compared to their respective counterparts. Additionally, those who were non-Whites and had less than high school education were more likely to express greater need for increased engagement in SDM in selecting the oral anticoagulant compared to their respective counterparts (Table 5).

In our sensitivity analysis stratified by sex, we found that among women, individuals from ethnic minority groups and those who reported greater burden from anticoagulation use were more likely to express a need for greater engagement in SDM when initiating anticoagulation compared to their male counterparts. However, in choosing the type of oral anticoagulant, there was no observed differences across the sex groups, as both women and men who were younger and expressed higher perceived burden from anticoagulation use, were more likely to prefer increased involvement in SDM (Supplement Material S1).

In the stratified analysis by the three multimorbidity groups, among those with 1–4 chronic conditions, younger participants (aged 65–74 years) and those with higher perceived anticoagulation burden indicated greater need for engagement in SDM on both initiating anticoagulation and choosing the anticoagulant. Among participants with 5–7 chronic conditions, higher perceived burden from anticoagulation use was associated with greater need for engagement in SDM for initiating anticoagulation and selecting anticoagulant type. Additionally, non-Whites and those with less than high school education were more likely to indicate a need for increased SDM on anticoagulant choice. Lastly, among participants with 8 or more chronic conditions, there were no significant findings of a need for greater engagement in SDM regarding initiating anticoagulation or choosing the anticoagulant (Supplement Material S1).

## DISCUSSION

In this cohort of older adults with AF and multimorbidity, we identified several subgroups of patients with the greatest need for involvement in stroke prevention decision making. One in three participants expressed preference for greater involvement in SDM for both initiating anticoagulation and selecting the oral anticoagulant for stroke prophylaxis. Younger participants (aged 65–74 years) and those with higher perceived burden from anticoagulation use were more likely to express greater need for increased SDM engagement initiating anticoagulation and selecting the anticoagulant. Additionally, non-Whites and those with less than high school education were more likely to indicate a stronger preference for increased involvement in SDM related to anticoagulant choice for stroke prevention. These findings were consistent among patients with fewer comorbidities in our stratified analysis.

### Need for Greater Engagement in SDM for Stroke Prevention in Varying Patient Subgroups

Although several SDM tools and decision aids have been designed to enhance patient-provider interactions for stroke prevention, our results underscore the need for greater engagement in SDM for older adults with AF and multimorbidity. This need is particularly pronounced in marginalized populations, including racial minority groups and individuals with lower levels of education, who face greater challenges navigating complex healthcare systems either due to poor access to care, lack of trust in physicians, and discrimination from clinicians [33,34]. Racial and ethnic minorities are most vulnerable to poorer engagement in healthcare decision-making leading to reduced decision satisfaction compared with their ethnic majority counterparts [35]. In addition, patients who are less educated, experience language barriers, or have low health literacy, are less knowledgeable about their treatment plan [35]. Our findings that older patients (75 years and older) were less likely to seek greater engagement in SDM, could be due to the “oldest-old” adults being more trusting of their clinicians’ recommendations and growing up in a paternalistic era where the doctor provided the treatment plan with little input from patients or caregivers. Future research is needed to better understand how the “oldest-old” adults would like to be engaged in SDM for stroke prevention.

In our sex-stratified analysis, women, especially those from ethnic minority backgrounds and those reporting a higher perceived burden from anticoagulation use expressed greater need for SDM in initiating anticoagulation compared to men. There were no sex differences in choosing the type of anticoagulant, as younger participants and those who experienced a higher burden from anticoagulation use across both sexes expressed a greater need for engagement in SDM. Previous research has demonstrated that women are less likely than men to receive anticoagulation therapy, despite facing a higher risk of stroke [15]. Present literature suggest that this discrepancy may be partly due concerns about a potentially increased bleeding risk in women with other cardiac conditions and comorbidities [36], despite prior research showing comparable major bleeding risk between men and women [37]. Our findings further suggest that women may be less actively engaged in the decision-making process regarding the initiation and selection of anticoagulation therapy. These results highlight the importance of acknowledging the unique challenges women face in managing AF and underscore the need for tailored, patient-centered approaches to their care.

Our study participants with a higher burden from anticoagulation use expressed greater need for SDM engagement initiating anticoagulation and selecting the anticoagulant. Enhancing involvement in SDM can help prepare patients and their caregivers to make informed decisions regarding anticoagulation therapy and proactively address challenges including monitoring requirements, lifestyle modifications, and side effects such as major bleeding. Future research should be geared towards developing innovative approaches to understanding patients’ unique needs and tailor complex and culturally sensitive discussions accordingly.

### Multimorbidity and Differential Need for Engagement in SDM

Most of our participants were diagnosed with 1–4 or 5–7 chronic conditions, while one in five participants had 8 or more chronic conditions. Our sensitivity analysis showed that patients with fewer comorbidities were more likely to express preference for greater engagement in SDM regarding initiating anticoagulation or selecting the oral anticoagulant. Conversely, those with 8 or more chronic conditions had no need for greater engagement in SDM regarding initiating anticoagulation or choosing the anticoagulant. Our paradoxical findings may be attributed to the challenges encountered by patients with greater number of comorbidities when managing their multiple conditions, making them less inclined to seek more engagement in SDM for their chronic diseases. This phenomenon can be attributed to “patient multimorbidity-burnout” which describes the myriads of psychological, emotional, and physical exhaustion that patients experience managing their comorbidities while simultaneously dealing with the cumulative burden of frequent doctor visits, complex medical regimens, treatment side effects, and the stress of living with multiple health problems [38,39]. Clinicians should promptly recognize this situation as patients with multimorbidity burnout may feel overwhelmed and be less inclined to actively participate in their care or adhere to treatment plans. Addressing patient multimorbidity-burnout involves better coordinated and holistic care that promotes engagement in SDM between patients, clinicians, and informal caregivers, simplifying and personalizing treatment regimens, taking into consideration cultural and spiritual factors that may influence healthcare engagement [40], as well as incorporating psychological and social support to improve patient physical, emotional, and overall well-being.

Furthermore, stroke prevention decisions in older adults with AF and multimorbidity is inherently complex requiring a careful balance between the risks of stroke and bleeding, considering overall life expectancy, managing competing health priorities, and aligning patient preferences [13,14]. These complexities highlight the importance of adopting personalized care models, such as Patient Priorities Care, that aligns treatment decisions with what matters most to patients, and may enhance SDM by addressing the health priorities, preferences, and treatment goals of older adults with AF and multimorbidity for optimal outcomes [41–43].

### Study Strengths and Limitations

A key strength of our study is its contribution of real-world evidence emphasizing the need for increased engagement in SDM for stroke prevention among older adults with AF and multimorbidity. This highlights an opportunity for clinicians to more effectively incorporate SDM into patient care, aligning with the latest guidelines from the AHA/HRS/ESC, which recommend SDM for decisions regarding anticoagulation in AF management [10,11].

Our study has some limitations. First, the use of an observational study design increases the risk of residual and unmeasured confounding, despite adjustment of a wide range of sociodemographic, psychosocial, and clinical characteristics. Second, there is a likelihood of recall bias as our survey items did not specify a timeline for engagement in SDM on stroke prevention. In addition, using single-item measures to assess patient preferences for greater engagement in SDM limits our ability to fully comprehend detailed insights into specific areas where patients may seek improvement in the decision-making process. For example, our findings of the need for more engagement in SDM may not directly correlate with actionable healthcare system factors but may be related to specific patient factors such as decreased health literacy or low social support. Future qualitative research, including in-depth interviews, is needed to explore how culturally sensitive and tailored personalized care models can enhance SDM engagement for older adults with AF and multiple chronic conditions.

## CONCLUSIONS

In managing older patients with AF and multimorbidity, there is need for a more comprehensive approach that facilitates SDM among healthcare providers, patients, and their caregivers to enhance understanding and treatment planning for stroke prevention. Our findings highlight the importance of clinicians prioritizing SDM, particularly among marginalized populations including racial minority groups, women, those with lower education levels, and patients with a higher burden of anticoagulation use for stroke prevention, to enhance patient-centered care and improve clinical outcomes. Clinicians should proactively identify patients at risk for multimorbidity-burnout and implement a holistic, coordinated care approach that includes the necessary support to encourage their active participation in SDM.

## Supplementary Material

Supplement Material S1

## Figures and Tables

**Table 1.** Study participants baseline characteristics.

Characteristics	*N* = 532

**Socio-demographic**	
Age (mean, years (sd))	75.0 (6.7)
Age categories (%)	
65–74 years	51.1
≥75 years	48.9
Women (%)	48.5
Race/Ethnicity (%)	
White	86.8
Non-White	13.2
Education	
≤high school	37.7
Some college	19.6
College graduate	42.7
**Psychosocial and Geriatric**	
Low social support (%)	23.5
Access to Internet Activity-last 4 weeks (%)	71.5
Visual Impairment (%)	32.7
Hearing Impairment (%)	33.3
Cognitive impairment (%)	31.7
Fair or poor self-rated health (%)	13.9
Frail (%)	10.9
Independent functioning (Mean, (SD))	6.8 (0.7)
**Clinical**	
AF Type (%)	
Paroxysmal	64.8
Persistent	16.9
Permanent	18.3
AF Treatment (%)	
Rhythm control	60.7
Rate control	39.3
Prior ablation therapy (%)	32.8
Anticoagulation therapy (%)	
Warfarin	56.2
Direct Acting Oral Anticoagulant	40.0
No anticoagulation prescribed	3.8
Polypharmacy (≥5 medications) (%)	28.2
CHA2DS2-VASc > 2 (%)	87.4
HASBLED risk score ≥ 3 (%)	73.8
**Patient Reported Outcomes**	
Confidence in Patient-Provider Interaction	
PEPPI score ≥45) (%)	71.4
Treatment Satisfaction with Anticoagulation	
ACTS Benefit Score (Mean, SD)	11.0 (3.8)
ACTS Burden Score (Mean, SD)	16.8 (5.8)

Abbreviations/Measures: CHA2DS2-VASc: Stroke risk assessment (Congestive heart failure, Hypertension, Age (≥65 = 1 point, ≥75 = 2 points), Diabetes, and prior Stroke/TIA (2 points), Vascular disease (peripheral arterial disease, previous MI, aortic atheroma) and female gender); HASBLED: Determines 1 year risk of major bleeding (Hypertension, Abnormal renal and liver function, prior Stroke, prior Bleeding, Labile INR, Elderly, Drugs or alcohol that increase risk of bleeding); Independent functioning assessed by Instrumental Activities of Daily Living (score range 0–7); SD: Standard Deviation.

**Table 2.** Patient characteristics according to need for engagement in SDM on initiating anticoagulation.

Characteristics	Patient Need for SDM on Initiating Anticoagulation Yes (*n* = 186)	Patient Need for SDM on Initiating Anticoagulation No (*n* = 346)	*p* Value

**Socio-demographic**			
Age (mean, years (sd))	73.8 (6.2)	75.6 (6.8)	**<0.01**
Age categories (%)			
65–74 years	58.6	47.1	**0.01**
≥75 years	41.4	52.9	
Women (%)	49.5	48.0	0.74
Non-Hispanic White	82.1	89.3	**0.02**
Non-White	17.9	10.7	
Education (%)			
≤high school	39.8	36.5	0.74
Some college	19.3	19.7	
College Graduate	40.9	43.8	
**Psychosocial and Geriatric**			
Low social support (%)	25.3	22.5	0.48
Access to Internet Activity-last 4	75.7	69.3	0.12
weeks (%)			
Visual Impairment (%)	37.6	30.1	0.08
Hearing Impairment (%)	32.8	33.5	0.86
Cognitive Impairment (%)	32.3	31.4	0.84
Fair or poor self-rated health (%)	14.5	13.6	0.77
Frail (%)	10.2	11.3	0.93
Independent functioning (Mean, (SD))	6.8 (0.6)	6.8 (0.7)	0.56
**Clinical**			
AF Type (%)			
Paroxysmal	67.4	63.4	0.17
Persistent	18.6	15.9	
Permanent	14.0	20.7	
AF Treatment (%)			
Rhythm control	61.3	60.4	0.84
Rate control	38.7	39.6	
Prior ablation therapy (%)	30.1	34.4	0.32
Anticoagulation therapy (%)			
Warfarin	54.3	57.2	0.16
Direct Acting Oral	39.8	40.2	
Anticoagulant			
No anticoagulation prescribed	5.9	2.6	
Polypharmacy (≥5 medications) (%)	32.3	26.0	0.13
CHA2DS2-VASc > 2 (%)	88.2	87.0	0.70
HASBLED risk score ≥ 3 (%)	72.6	74.6	0.62
**Patient Reported Outcomes**			
Confidence in Patient-Provider			
Interaction			
PEPPI score ≥ 45 (%)	64.3	75.1	**0.01**
Treatment Satisfaction with			
Anticoagulation			
ACTS Benefit Score (Mean, SD)	10.6 (3.7)	11.2 (3.8)	0.06
ACTS Burden Score (Mean, SD)	18.4 (6.8)	15.9 (5.0)	**<0.001**
Current Smoker	1.6	2.6	

Abbreviations/Measures: CHA2DS2-VASc: Stroke risk assessment (Congestive heart failure, Hypertension, Age (≥65 = 1 point, ≥75 = 2 points), Diabetes, and prior Stroke/TIA (2 points), Vascular disease (peripheral arterial disease, previous MI, aortic atheroma) and female gender); HASBLED: Determines 1 year risk of major bleeding (Hypertension, Abnormal renal and liver function, prior Stroke, prior Bleeding, Labile INR, Elderly, Drugs or alcohol that increase risk of bleeding); Independent functioning assessed by Instrumental Activities of Daily Living (score range 0–7); The bold text indicates statistically significant results with *p* < 0.05.

**Table 3.** Patient characteristics according to need for engagement in SDM in choosing anticoagulation type.

Characteristics	Patient Need for SDM in Choosing Anticoagulation Type Yes (*n* = 175)	Patient Need for SDM in Choosing Anticoagulation Type No (*n* = 357)	*p* Value

**Socio-demographic**			
Age (mean, years (sd))	73.2 (5.7)	75.8 (6.9)	**<0.001**
Age categories (%)			
65–74 years	62.9	45.4	**<0.001**
≥75 years	37.1	54.6	
Women (%)	45.7	49.8	0.37
Non-Hispanic White	79.3	90.5	**<0.001**
Non-White	20.7	9.5	
Education (%)			
≤high school	42.3	35.4	0.15
Some college	15.4	21.6	
College Graduate	42.3	43.0	
**Psychosocial and Geriatric**			
Low social support (%)	27.4	21.6	0.13
Access to Internet Activity-last 4 weeks (%)	73.1	70.7	0.56
Visual Impairment (%)	37.7	30.2	0.08
Hearing Impairment (%)	32.6	33.6	0.81
Cognitive impairment (%)	30.9	32.1	0.77
Fair or poor self-rated health (%)	18.3	11.8	**0.04**
Frail (%)	12.0	10.4	0.85
Independent functioning (Mean, (SD))	6.9 (0.4)	6.8 (0.8)	0.09
**Clinical**			
AF Type (%)			
Paroxysmal	66.3	64.1	0.45
Persistent	18.4	16.1	
Permanent	15.3	19.8	
AF Treatment (%)			
Rhythm control	63.4	59.4	0.37
Rate control	36.6	40.6	
Prior ablation therapy (%)	29.7	34.5	0.27
Anticoagulation therapy (%)			
Warfarin	56.0	56.3	0.49
Direct Acting Oral Anticoagulant	38.9	40.6	
No anticoagulation prescribed	5.1	3.1	
Polypharmacy (≥5 medications) (%)	32.6	26.1	0.12
CHA2DS2-VASc > 2 (%)	87.4	87.4	0.99
HASBLED risk score ≥ 3 (%)	70.9	75.6	0.27
**Patient Reported Outcomes**			
Confidence in Patient-Provider Interaction			
PEPPI score ≥ 45 (%)	66.9	73.6	0.11
Treatment Satisfaction with Anticoagulation			
ACTS Benefit Score (Mean, SD)	10.4 (3.5)	11.3 (3.9)	**0.02**
ACTS Burden Score (Mean, SD)	18.8 (6.9)	15.8 (4.9)	**<0.001**

Abbreviations/Measures: CHA2DS2-VASc: Stroke risk assessment (Congestive heart failure, Hypertension, Age (≥65 = 1 point, ≥75 = 2 points), Diabetes, and prior Stroke/TIA (2 points), Vascular disease (peripheral arterial disease, previous MI, aortic atheroma) and female gender); HASBLED: Determines 1 year risk of major bleeding (Hypertension, Abnormal renal and liver function, prior Stroke, prior Bleeding, Labile INR, Elderly, Drugs or alcohol that increase risk of bleeding); Independent functioning assessed by Instrumental Activities of Daily Living (score range 0–7); The bold text indicates statistically significant results with *p* < 0.05.

**Table 4.** Factors associated with patient preference for engagement in SDM for initiation of anticoagulation for stroke prevention.

Participant Characteristics	Crude Model OR (95% CI)	Multivariable Model OR (95% CI)

Age		
≥75 years	Ref	Ref
65–74 years	1.58 (1.11–2.28)	**1.53 (1.03–2.27)**
Race/Ethnicity		
Non-Hispanic White	Ref	Ref
Non-White	1.82 (1.10–3.03)	1.58 (0.89–2.81)
Education (%)		
Some college	Ref	Ref
≤High school	1.11 (0.67–1.82)	1.02 (0.59–1.76)
College Graduate	0.95 (0.58–1.55)	1.02 (0.61–1.71)
Confidence in Patient-Provider Interaction (PEPPI > 45)	0.59 (0.40–0.88)	0.72 (0.47–1.10)
Patient report of anticoagulation benefit (ACTS Score)	0.95 (0.91–1.00)	0.97 (0.93–1.03)
Patient report of anticoagulation burden (ACTS Score)	1.07 (1.04–1.11)	**1.06 (1.03–1.10)**

The bold text indicates statistically significant results with *p* < 0.05.

**Table 5.** Factors associated with patient preference for engagement in SDM for type of anticoagulation for stroke prevention.

Participant Characteristics	Crude Model OR (95% CI)	Multivariable Model OR (95% CI)

Age		
≥75 years	Ref	Ref
65–74 years	2.04 (1.41–2.95)	**2.02 (1.34–3.06)**
Race/Ethnicity		
Non-Hispanic White	Ref	Ref
Non-White	2.47 (1.48–4.11)	**1.92 (1.05–3.49)**
Education (%)		
Some college	Ref	Ref
≤High school	1.67 (1.00–2.83)	**1.91 (1.06–3.44)**
College Graduate	1.38 (0.82–2.32)	1.63 (0.93–2.87)
Low self-rated health	1.68 (1.02–2.77)	1.05 (0.58–1.90)
Patient report of anticoagulation benefit (ACTS Score)	0.95 (0.90–0.99)	0.97 (0.92–1.03)
Patient report of anticoagulation burden (ACTS Score)	1.09 (1.06–1.13)	**1.09 (1.05–1.13)**

The bold text indicates statistically significant results with *p* < 0.05.

## HIGHLIGHTS

- One in three older adults with atrial fibrillation and multimorbidity had a need for greater engagement in shared decision-making for stroke prevention regarding the choice to be on anticoagulation and deciding on the type of oral anticoagulant.
- Patients who were younger (aged 65–74 years), non-White, less educated, or reported a higher perceived burden associated with anticoagulation use were more likely to report the need for greater engagement in shared decision-making for stroke prevention.
- Among women, particularly those from ethnic minority groups and those experiencing a higher perceived burden from anticoagulation use expressed a greater need for shared decision making in initiating anticoagulation compared to their male counterparts.
- Patients with fewer comorbidities were more likely to have a need for greater engagement in shared decision-making for stroke prevention compared to those with 8 or more comorbidities, which may be suggestive of patient-multimorbidity burnout.

## WHY DOES THIS PAPER MATTER?

Healthcare providers should acknowledge the unique needs of older adults with AF and multiple chronic conditions who desire greater involvement in SDM for stroke prevention. Implementing tailored interventions can enhance engagement in the decision-making process for stroke prevention in this vulnerable population, ultimately leading to improved patient-centered and clinical outcomes.

## ABBREVIATIONS

ACTS: AntiCoagulation Treatment Satisfaction
AF: Atrial Fibrillation
AHA: American Heart Association
CHS: Cardiovascular Health Survey
CI: Confidence Interval
DOAC: Direct acting Oral AntiCoagulation
EMR: Electronic Medical Record
ESC: European Society of Cardiology
HRS: Heart Rhythm Society
IADLs: Instrumental Activities of Daily Living
MA: Massachusetts
MOCA: Montreal Cognitive Assessment
OR: Odds Ratio
PEPPI: Perceived Efficacy in Patient-Physician Interactions
SAGE-AF: Systematic Assessment of Geriatric Elements in Atrial Fibrillation
SDM: Shared Decision-Making
US-DHHS: U.S. Department of Health and Human Services
