# Supplementary material for: Need for Engagement in Stroke Prevention Shared Decision-Making in Older Adults with Atrial Fibrillation and Multimorbidity
Source: Adv Geriatr Med Res. Author manuscript; Available in PMC 2025 Dec 18. (PMC12710876; doi:10.20900/agmr20250016)
Supplement: Supplement Material S1 [file NIHMS2101553-supplement-Supplement_Material_S1.pdf]

SUPPLEMENT MATERIAL S1: STRATIFIED ANALYSIS BY SEX AND MULTIMORBIDITY GROUPS

Table S1. Factors associated with patient preference for engagement in SDM for initiation of anticoagulation for stroke prevention (Women, *n* = 242).

Participant Characteristics	Crude Model OR (95% CI)	Multivariable Model OR (95% CI)
Age		
≥75 years	Ref	Ref
65–74 years	2.13 (01.27–3.59)	1.58 (0.87–2.86)
Race/Ethnicity		
Non-Hispanic White	Ref	Ref
Non-White	2.70 (1.25–5.86)	**2.90 (1.16–7.26)**
Education (%)		
Some college	Ref	Ref
≤High school	0.82 (0.42–1.58)	0.77 (0.36–1.64)
College Graduate	0.85 (0.43–1.66)	0.98 (0.46–2.08)
Multimorbidity categories		
1–4	Ref	Ref
5–7	1.23 (0.69–0.56)	1.45 (0.76–2.77)
8 or more	1.12 (0.56–2.23)	1.22 (0.56–2.65)
Confidence in Patient-Provider Interaction (PEPPI > 45)	0.61 (0.35–1.07)	0.89 (0.47–1.67)
Patient report of anticoagulation benefit (ACTS Score)	0.95 (0.89–1.02)	0.99 (0.91–1.07)
Patient report of anticoagulation burden (ACTS Score)	1.09 (1.04–1.14)	**1.08 (1.03–1.14)**

Abbreviations: PEPPI: Perceived Efficacy in Patient-Physician Interactions; ACTS: AntiCoagulation Treatment Satisfaction. Bold text indicates statistically significant results with $p < 0.05$.

Table S2. Factors associated with patient preference for engagement in SDM for initiation of anticoagulation for stroke prevention (Men, *n* = 253).

Participant Characteristics	Crude Model OR (95% CI)	Multivariable Model OR (95% CI)
Age		
≥75 years	Ref	Ref
65–74 years	1.21 (0.73–2.00)	1.35 (0.77–2.34)
Race/Ethnicity		
Non-Hispanic White	Ref	Ref
Non-White	1.35 (0.68–2.69)	0.99 (0.45–2.19)
Education (%)		
Some college	Ref	Ref
≤High school	1.64 (0.76–3.52)	1.41 (0.62–3.20)
College Graduate	1.18 (0.56–2.46)	1.15 (0.53–2.49)
Multimorbidity categories		
1–4	Ref	Ref
5–7	1.08 (0.62–1.89)	0.95 (0.51–1.74)
8 or more	1.26 (0.63–2.53)	1.04 (0.49–2.20)
Confidence in Patient-Provider Interaction (PEPPI > 45)	0.58 (0.33–1.00)	0.23 (0.34–1.12)
Patient report of anticoagulation benefit (ACTS Score)	0.96 (0.89–1.02)	0.97 (0.90–1.04)
Patient report of anticoagulation burden (ACTS Score)	1.05 (1.00–1.11)	1.03 (0.98–1.09)

Abbreviations: PEPPI: Perceived Efficacy in Patient-Physician Interactions; ACTS: AntiCoagulation Treatment Satisfaction.

Table S3. Factors associated with patient preference for engagement in SDM for type of anticoagulant for stroke prevention (Women, n = 248).

Participant Characteristics	Crude Model OR (95% CI)	Multivariable Model OR (95% CI)
Age		
≥75 years	Ref	Ref
65–74 years	2.61 (1.51–4.51)	**2.06 (1.09–3.89)**
Race/Ethnicity		
Non-Hispanic White	Ref	Ref
Non-White	2.91 (1.34–6.31)	2.44 (0.94–6.31)
Education (%)		
Some college	Ref	Ref
≤High school	1.34 (0.66–2.71)	1.92 (0.85–4.37)
College Graduate	1.20 (0.58–2.47)	1.48 (0.64–3.42)
Multimorbidity categories		
1–4	Ref	Ref
5–7	1.03 (0.57–1.85)	0.92 (0.47–1.79)
8 or more	0.68 (0.32–1.43)	0.64 (0.27–1.51)
Low self-rated health	2.18 (1.03–4.63)	1.39 (0.55–3.55)
Patient report of anticoagulation benefit (ACTS Score)	0.95 (0.89–1.02)	0.99 (0.92–1.08)
Patient report of anticoagulation burden (ACTS Score)	1.12 (1.07–1.17)	**1.11 (1.05–1.17)**

Abbreviations: ACTS: AntiCoagulation Treatment Satisfaction. Bold text indicates statistically significant results with $p < 0.05$.

Table S4. Factors associated with patient preference for engagement in SDM for type of anticoagulant for stroke prevention (Men, n = 257).

Participant Characteristics	Crude Model OR (95% CI)	Multivariable Model OR (95% CI)
Age		
≥75 years	Ref	Ref
65–74 years	1.62 (0.98–2.69)	**1.81 (1.03–3.19)**
Race/Ethnicity		
Non–Hispanic White	Ref	Ref
Non–White	2.15 (1.09–4.23)	1.64 (0.75–3.60)
Education (%)		
Some college	Ref	Ref
≤High school	2.15 (0.97–4.76)	1.91 (0.80–4.53)
College Graduate	1.58 (0.73–3.41)	1.61 (0.72–3.61)
Multimorbidity categories		
1–4	Ref	Ref
5–7	1.00 (0.57–1.74)	0.88 (0.47–1.62)
8 or more	1.31 (0.66–2.62)	1.04 (0.46–2.32)
Low self-rated health	1.32 (0.68–2.63)	0.86 (0.37–1.99)
Patient report of anticoagulation benefit (ACTS Score)	0.94 (0.88–1.01)	0.96 (0.89–1.03)
Patient report of anticoagulation burden (ACTS Score)	1.07 (1.01–1.12)	**1.06 (1.01–1.12)**

Abbreviations: ACTS: AntiCoagulation Treatment Satisfaction. Bold text indicates statistically significant results with $p < 0.05$.

Table S5. Factors associated with patient preference for engagement in SDM for initiation of anticoagulation for stroke prevention (1–4 chronic conditions, n = 203).

**Participant Characteristics**	**Crude Model OR (95% CI)**	**Multivariable Model OR (95% CI)**
Age		
≥75 years	Ref	Ref
65–74 years	2.09 (1.15–3.78)	**2.05 (1.07–3.94)**
Race/Ethnicity		
Non-Hispanic White	Ref	Ref
Non-White	2.19 (0.79–6.11)	1.62 (0.48–5.39)
Education (%)		
Some college	Ref	Ref
≤High school	0.87 (0.38–1.98)	0.61 (0.24–1.56)
College Graduate	0.93 (0.43–2.00)	0.93 (0.41–2.09)
Confidence in Patient-Provider Interaction (PEPPI > 45)	0.75 (0.40–1.41)	0.81 (0.41–1.60)
Patient report of anticoagulation benefit (ACTS Score)	0.93 (0.86–1.01)	0.94 (0.86–1.02)
Patient report of anticoagulation burden (ACTS Score)	1.07 (1.01–1.13)	**1.06 (1.00–1.13)**

Abbreviations: PEPPI: Perceived Efficacy in Patient-Physician Interactions; ACTS: AntiCoagulation Treatment Satisfaction. Bold text indicates statistically significant results with $p < 0.05$.

Table S6. Factors associated with patient preference for engagement in SDM for initiation of anticoagulation for stroke prevention (5–7 chronic conditions, n = 193).

**Participant Characteristics**	**Crude Model OR (95% CI)**	**Multivariable Model OR (95% CI)**
Age		
≥75 years	Ref	Ref
65–74 years	1.10 (0.63–1.93)	1.43 (0.73–2.80)
Race/Ethnicity		
Non–Hispanic White	Ref	Ref
Non–White	1.61 (0.76–3.42)	1.23 (0.51–2.95)
Education (%)		
Some college	Ref	Ref
≤High school	1.71 (0.76–3.87)	2.07 (0.83–5.19)
College Graduate	0.88 (0.38–2.00)	0.92 (0.37–2.26)
Confidence in Patient-Provider Interaction (PEPPI > 45)	0.50 (0.27–0.93)	0.65 (0.33–1.29)
Patient report of anticoagulation benefit (ACTS Score)	0.95 (0.88–1.02)	0.95 (0.88–1.03)
Patient report of anticoagulation burden (ACTS Score)	1.07 (1.02–1.13)	**1.07 (1.01–1.12)**

Abbreviations: PEPPI: Perceived Efficacy in Patient-Physician Interactions; ACTS: AntiCoagulation Treatment Satisfaction. Bold text indicates statistically significant results with $p < 0.05$.

Table S7. Factors associated with patient preference for engagement in SDM for initiation of anticoagulation for stroke prevention (≥8 chronic conditions, n = 99).

Participant Characteristics	Crude Model OR (95% CI)	Multivariable Model OR (95% CI)
Age		
≥75 years	Ref	Ref
65–74 years	1.90 (0.84–4.29)	1.48 (0.59–3.71)
Race/Ethnicity		
Non-Hispanic White	Ref	Ref
Non-White	(0.66–4.63)	2.03 (0.66–6.30)
Education (%)		
Some college	Ref	Ref
≤High school	0.73 (0.27–1.97)	0.68 (0.22–2.09)
College Graduate	1.30 (0.44–3.82)	1.48 (0.47–4.59)
Confidence in Patient-Provider Interaction (PEPPI > 45)	0.57 (0.24–1.36)	0.74 (0.28–1.94)
Patient report of anticoagulation benefit (ACTS Score)	1.02 (0.91–1.13)	1.06 (0.93–1.21)
Patient report of anticoagulation burden (ACTS Score)	1.08 (1.00–1.16)	1.07 (0.99–1.16)

Abbreviations: PEPPI: Perceived Efficacy in Patient-Physician Interactions; ACTS: AntiCoagulation Treatment Satisfaction.

Table S8. Factors associated with patient preference for engagement in SDM for type of anticoagulant for stroke prevention (1–4 chronic conditions, n = 206).

Participant Characteristics	Crude Model OR (95% CI)	Multivariable Model OR (95% CI)
Age		
≥75 years	Ref	Ref
65–74 years	2.52 (1.37–4.61)	**2.36 (1.21–4.61)**
Race/Ethnicity		
Non-Hispanic White	Ref	Ref
Non-White	3.74 (1.30–10.76)	2.11 (0.61–7.27)
Education (%)		
Some college	Ref	Ref
≤High school	1.50 (0.64–3.50)	1.32 (0.49–3.53)
College Graduate	1.32 (0.59–2.94)	1.44 (0.59–3.48)
Low self-rated health	2.84 (1.01–7.98)	1.90 (0.53–6.82)
Patient report of anticoagulation benefit (ACTS Score)	0.94 (0.88–1.01)	0.95 (0.87–1.04)
Patient report of anticoagulation burden (ACTS Score)	1.13 (1.06–1.20)	**1.12 (1.04–1.20)**

Abbreviations: ACTS: AntiCoagulation Treatment Satisfaction. Bold text indicates statistically significant results with $p < 0.05$.

Table S9. Factors associated with patient preference for engagement in SDM for type of anticoagulant for stroke prevention (5–7 chronic conditions, n = 199).

Participant Characteristics	Crude Model OR (95% CI)	Multivariable Model OR (95% CI)
Age		
≥75 years	Ref	Ref
65–74 years	1.41 (0.79–2.51)	1.63 (0.82–3.25)
Race/Ethnicity		
Non-Hispanic White	Ref	Ref
Non-White	3.42 (1.59–7.34)	**3.10 (1.27–7.58)**
Education (%)		
Some college	Ref	Ref
≤High school	2.20 (0.92–5.26)	**2.85 (1.04–7.78)**
College Graduate	1.31 (0.55–3.15)	1.62 (0.61–4.35)
Low self-rated health	1.86 (0.81–4.28)	1.20 (0.45–3.21)
Patient report of anticoagulation benefit (ACTS Score)	0.96 (0.89–1.04)	0.98 (0.90–1.07)
Patient report of anticoagulation burden (ACTS Score)	1.08 (1.03–1.13)	**1.10 (1.04–1.16)**

Abbreviations: ACTS: AntiCoagulation Treatment Satisfaction. Bold text indicates statistically significant results with $p < 0.05$.

Table S10. Factors associated with patient preference for engagement in SDM for type of anticoagulant for stroke prevention (≥8 chronic conditions, n = 100).

Participant Characteristics	Crude Model OR (95% CI)	Multivariable Model OR (95% CI)
Age		
≥75 years	Ref	Ref
65–74 years	2.67 (1.12–6.33)	2.29 (0.88–5.95)
Race/Ethnicity		
Non-Hispanic White	Ref	Ref
Non-White	1.08 (0.39–2.98)	0.88 (0.27–2.90)
Education (%)		
Some college	Ref	Ref
≤High school	1.23 (0.43–3.56)	1.40 (0.44–4.45)
College Graduate	1.66 (0.53–5.22)	2.13 (0.63–7.21)
Low self-rated health	1.17 (0.48–2.83)	0.84 (0.29–2.44)
Patient report of anticoagulation benefit (ACTS Score)	0.93 (0.84–1.04)	0.96 (0.84–1.09)
Patient report of anticoagulation burden (ACTS Score)	1.07 (0.99–1.14)	1.06 (0.98–1.15)

Abbreviations: ACTS: AntiCoagulation Treatment Satisfaction.
